# Pharmacokinetics of a Long-Acting Nanoformulated Dolutegravir Prodrug in Rhesus Macaques

**DOI:** 10.1128/AAC.01316-17

**Published:** 2017-12-21

**Authors:** JoEllyn McMillan, Adam Szlachetka, Lara Slack, Brady Sillman, Benjamin Lamberty, Brenda Morsey, Shannon Callen, Nagsen Gautam, Yazen Alnouti, Benson Edagwa, Howard E. Gendelman, Howard S. Fox

**Affiliations:** a Pharmacology and Experimental Neuroscience, University of Nebraska Medical Center, Omaha, Nebraska, USA; b Nebraska Nanomedicine Production Plant, University of Nebraska Medical Center, Omaha, Nebraska, USA; c Pharmaceutical Sciences, University of Nebraska Medical Center, Omaha, Nebraska, USA

**Keywords:** dolutegravir, long-acting antiretrovirals, monkeys, nanoformulated antiretrovirals

## Abstract

A nanoformulated myristoylated dolutegravir prodrug (NMDTG) was prepared using good laboratory practice protocols. Intramuscular injection of NMDTG (118 ± 8 mg/ml, 25.5 mg of DTG equivalents/kg of body weight) to three rhesus macaques led to plasma DTG levels of 86 ± 12 and 28 ± 1 ng/ml on days 35 and 91, respectively. The NMDTG platform showed no significant adverse events. Further modification may further extend the drug's apparent half-life for human use.

## TEXT

Antiretroviral drug (ARV) therapy allows those infected with human immunodeficiency virus (HIV) to live normal productive lives (1, 2). Nonetheless, adherence to lifelong daily multidrug regimens and viral resistance continue (3–8). End-organ disease, drug-drug interactions, cost, short pharmacologic duration, and access are further limitations. We posit that long-acting ARVs can overcome these restrictions (7, 9–12). In a step to transform existing ARVs into long-acting medicines, we increased the hydrophobicity and lipophilicity of ARVs through carboxy-ester myristoylation, affecting tissue and cell penetrance (13, 14). In the current report, this approach was developed for dolutegravir (DTG), a second-generation integrase strand transfer inhibitor with high viral resistance barriers (15, 16). Native orally administered DTG demonstrates a plasma half-life of 13 to 14 h (17–19), with >99% plasma albumin and alpha 1-acid glycoprotein binding. A daily or twice-daily dosing is required to maintain therapeutic plasma concentrations above a protein-adjusted 90% inhibitory concentration (PA-IC_90_) of 64 ng/ml (15, 17–19). To extend the pharmacokinetic properties of DTG, a nanoformulated prodrug derivative (nanoformulated myristoylated dolutegravir [NMDTG]) was made. Intramuscular injections with 45 mg/kg of body weight led to drug levels in mice above the PA-IC_90_ for 56 days (our unpublished data) and were then affirmed in nonhuman primates.

Myristoylated DTG (MDTG) was prepared by a one-step synthesis and then encased into a nanoformulation (our unpublished data). Synthesis and quality control measures were maintained through the Nebraska Nanomedicine Production Plant using good laboratory practice (GLP) protocols following U.S. Food and Drug Administration (FDA) guidelines (20) (see the supplemental material). The prepared suspensions were homogenized at 20,000 lb/in^2^ using an Avestin EmulsiFlex-C3 high-pressure homogenizer (Avestin, Inc., Ottawa, Ontario, Canada). Particle sizes, polydispersity indices, and zeta potentials were determined by dynamic light scattering (Malvern Zetasizer Nano ZSP; Malvern Instruments, Westborough, MA). MDTG concentrations in the nanosuspensions were determined by ultraperformance liquid chromatography-tandem mass spectrometry (UPLC-MS/MS; Waters Acquity XevoTQ-S micro system; Waters Corp., Milford, MA) (see supplemental material). Endotoxin content was determined by Lonza Limulus Amebocyte Lysate Pyrogent-500 tests (Lonza, Walkersville, MD). Animal studies were conducted in accordance with the University of Nebraska Medical Center Institutional Animal Care and Use Committee, according to federal guidelines. Three male rhesus macaques (9 to 10 kg; purchased from PrimeGen) were anesthetized with 10 mg/kg ketamine and injected intramuscularly (i.m.) with undiluted NMDTG suspension (38.5 mg/kg in 2.67 to 3.50 ml). Blood was collected into potassium-EDTA tubes before NMDTG administration, days 1, 4, and 7 after administration, and weekly thereafter to day 91. Plasma and peripheral blood mononuclear cells (PBMCs) were obtained for complete blood counts, metabolic panels, and DTG and MDTG drug quantitation. These were performed by the Nebraska Medical Center Pathology and Microbiology laboratory and by the Nebraska Nanomedicine Production Plant GLP laboratory using UPLC-MS/MS, respectively (see supplemental material). Animal health and injection site reactions were monitored daily. NMDTG was manufactured in three separate batches with reproducible characteristics, as summarized in Table 1. Nanoparticle sizes varied from 275.8 to 338.6 nm, with a narrow polydispersity index (0.20 to 0.25) and a negative zeta potential (−18.9 to −22.3 mV). MDTG concentrations in the nanosuspensions were from 111.9 to 126.6 mg/ml. The DTG concentration was below the limit of detection (0.2 ng/ml). To ensure aseptic preparation of the nanosuspensions, the endotoxin levels in the prepared nanoformulations were determined. Formulation endotoxin concentrations were below 5 endotoxin units (EU)/kg.

**TABLE 1:** Physicochemical characteristics of NMDTG batches

Characteristic	Results by animal^*a*^			Avg
	5009	6039	6049	
Particle size (nm)	338.6	314.8	275.8	309.7 ± 31.7
PDI^*b*^	0.25	0.20	0.20	0.22 ± 0.03
Zeta potential (mV)	−22.3	−19.8	−18.9	−20.3 ± 1.8
MDTG concn (mg/ml)	111.9	115.6	126.6	118.0 ± 7.6

a Animals are those for which each formulation batch was prepared.

b PDI, polydispersity index.

**FIG 1:**
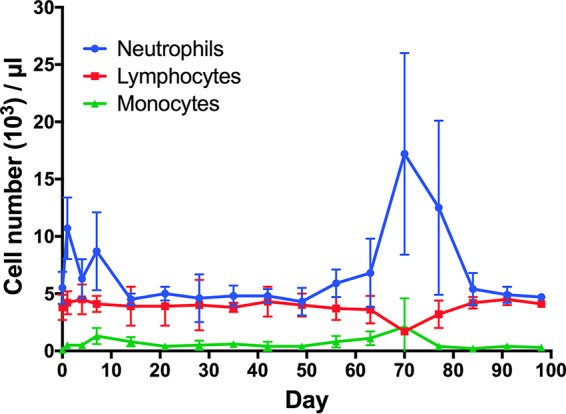
White blood cell counts following NMDTG administration in rhesus macaques. Complete blood count (CBC) samples were collected into EDTA tubes and assessed by manual differentiation. Data are expressed as mean ± standard deviation (*n* = 3).

**FIG 2:**
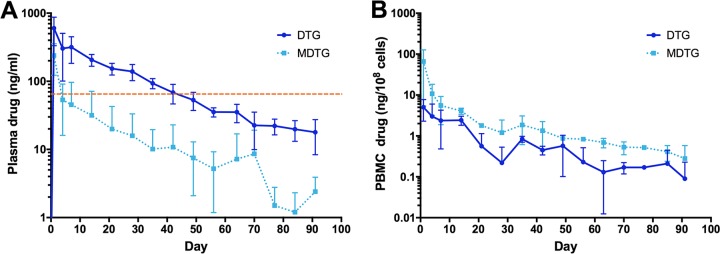
Plasma (A) and blood leukocyte (B) DTG and MDTG concentrations following NMDTG administration in rhesus macaques. Data are expressed as mean ± standard deviation (A, *n* = 3; B, *n* = 2 to 3). Dashed line indicates DTG PA-IC_90_ of 64 ng/ml.

A single i.m. injection of NMDTG was administered to three rhesus macaques. Neutrophil, lymphocyte, and monocyte counts determined prior to NMDTG administration and weekly after administration (Fig. 1) were consistent. A solitary increase in neutrophil counts was recorded at day 70 and reflected the development of an abscess near the site of injection in a single animal and was deemed not to be drug associated. Full resolution was made by drainage and antibiotic treatment. An initial mild redness and swelling observed at the injection site were resolved in all animals by day 7. Liver and kidney metabolic profiles were unchanged in all animals following NMDTG treatment (see Tables S1 to S3 in the supplemental material). No weight changes or adverse physical signs were recorded in any of the animals after formulation injection (data not shown). Parent and prodrug concentrations in plasma and blood leukocytes were determined following NMDTG administration. Plasma DTG concentrations peaked at day 1 at 602 ± 269 ng/ml and remained above the protein-adjusted 90% inhibitory concentration (PA-IC_90_) of 64 ng/ml for 35 days (Fig. 2A) and above 10 ng/ml for 91 days. The extended pharmacokinetic profile of NMDTG was demonstrated by the assessment of DTG plasma half-life (*t*_1/2_; 467 ± 28 h) and mean residence time (MRT; 691.7 ± 98.4 h) (Table 2). Plasma prodrug concentrations, although varied, remained above the limit of detection (at or around 10 ng/ml) for 42 days. MDTG was observed in blood leukocytes (Fig. 2B) through day 42; however, DTG concentrations were at the limit of detection after 14 days. The pharmacokinetic (PK) parameters of both MDTG and DTG in PBMCs reflected those in plasma, especially the half-lives, which indicates that the intracellular and plasma concentrations were in equilibrium. Similar to plasma, an extended half-life was observed for both MDTG (*t*_1/2_ = 542 h) and DTG (*t*_1/2_= 460 h) in PBMCs (Table 3).

**TABLE 2:** PK parameters for DTG and MDTG

PK parameter^*a*^	DTG^*b*^		MDTG	
	Avg	SEM	Avg	SEM
λ_Z_ (h^−1^)	0.0015	0.0001	0.0016	0.0002
*t*_1/2_ (h)	467.1	28.1	458.2	55.7
AUC_last_ (h · ng/ml)	235,061.6	33,152.3	40,864.0	16,260.0
AUC_0–∞_ (h · ng/ml)	249,640.2	35,707.7	41,937.7	16,271.1
*V*_β_/*F* (liters/kg)	ND	—	835.5	334.0
CL/*F* (liters/h/kg)	ND	—	1.19	0.36
MRT_0–∞_ (h)	691.7	98.4	454.6	62.5

a In the units of measure for the area under the concentration-time curve (AUC) data, 1 ml is equivalent to 100 × 10^6^ cells. λ_Z_, individual estimate of the terminal elimination rate constant; AUC_last_, AUC 0 h to last time point; AUC_0–∞_, AUC from 0 h to infinity; *V*_β_, volume of distribution at β phase; CL, clearance; MRT, mean residence time. PK parameters are calculated from the mean of the results from 3 monkeys.

b SEM, standard error of the mean; ND, not determined.

**TABLE 3:** PBMC PK parameters for DTG and MDTG

PK parameter^*a*^	DTG	MDTG
λ_Z_ (h^−1^)	0.0015	0.0013
*t*_1/2_ (h)	460	542
AUC_last_ (h · ng/ml)	1,755	7,009
AUC_0–∞_ (h · ng/ml)	1,815	7,228
MRT_0–∞_ (h)	571	409

a In the units of measure for the AUC data, 1 ml is equivalent to 100 × 10^6^ cells. λ_Z_, individual estimate of the terminal elimination rate constant; AUC_last_, AUC 0 h to last time point; AUC_0–∞_, AUC from 0 h to infinity; *V*_β_, volume of distribution at β phase; CL, clearance; MRT, mean residence time. PK parameters are calculated from the mean of the results from 3 monkeys.

The idea of long-acting injectable drugs for the chronic treatment of disease has found therapeutic precedent in the areas of antipsychotics and contraception (21–24). The development of long-acting injectables for antiretroviral drugs has great promise. This includes, but is not limited to, the potential to improve regimen adherence, reduction of drug side effects, and reduce the development of drug resistance (10, 25), as well as the potential to serve as a preexposure prophylaxis regimen (10, 17, 21). To prove practical for human use, the medicines need to provide prophylactic prevention of human immunodeficiency virus type 1 (HIV-1) infection when given at intervals of once a month or longer (10, 11, 17). Our laboratories have been at the forefront of this effort by using chemical modifications of hydrophilic and hydrophobic drugs to improve drug hydrophobicity and lipophilicity (13, 14). Incorporation of the hydrophobic lipophilic derivatives into nanoformulations has produced injectable formulations of drugs that can be administered monthly to target viral cell and tissue reservoirs and suppress HIV infection in humanized mouse models (13, 14, 26). The present data demonstrate that a single i.m. injection of long-acting NMDTG provides plasma drug levels above the PA-IC_90_ in rhesus macaques for 1 month and can greatly extend drug half-life. The relatively higher MDTG levels compared to those of DTG in PBMCs indicate that the nanoformulation is taken into mononuclear phagocytes that can act as secondary drug depots, as we have observed in rodent studies (27, 28). The study illustrates that these formulations can be reproducibly and safely produced, and with additional modest modifications in volume and in hydrophobicity, they could be ready for human clinical trial scale-ups. This is a major step forward in the development and production of a long-acting DTG formulation. Additional drug and formulation modifications are in development to effectively realize a translatable formulation that can be delivered in a clinically relevant dose volume and provide, following a single injection, plasma drug levels at 1 month above the trough concentration (*C*_trough_) level of 840 ng/ml observed in adults given a 50-mg daily oral dose of DTG (19).

## Supplementary Material

Supplemental material
