# Supplementary material for: Pharmacokinetics of a Long-Acting Nanoformulated Dolutegravir Prodrug in Rhesus Macaques
Source: Antimicrob Agents Chemother. 2017 Dec 21;62(1):e01316-17. doi: 10.1128/AAC.01316-17 (PMC5740312; doi:10.1128/AAC.01316-17)
Supplement: Supplemental material [file supp_62_1_e01316-17__index.html]

Pharmacokinetics of a Long-Acting Nanoformulated Dolutegravir Prodrug in Rhesus Macaques — Supplemental material 

# Pharmacokinetics of a Long-Acting Nanoformulated Dolutegravir Prodrug in Rhesus Macaques

## Supplemental material

- Supplemental file 1 -

  Supplemental text and tables

  PDF, 187K
