# Supplementary material for: Pharmacokinetics of a Long-Acting Nanoformulated Dolutegravir Prodrug in Rhesus Macaques
Source: Antimicrob Agents Chemother. 2017 Dec 21;62(1):e01316-17. doi: 10.1128/AAC.01316-17 (PMC5740312; doi:10.1128/AAC.01316-17)
Supplement: Supplemental material [file AAC.01316-17_zac001186783s1.pdf]

SUPPLEMENTAL TEXT

Nanomedicine Production Plant Good Laboratory Practice (GLP) Facility Production of NMDTG

The Operational Unit and Quality Assurance Unit work together, but independently of each other, to put into place the protocols and guidelines, which were followed during the manufacturing of NMDTG in the Good Laboratory Practice (GLP) Facility.

The Operational Unit was responsible for following approved protocols and standard operating procedures (SOPs). All of the equipment followed strict protocols for calibration and cleaning before and after use. A witness verified all procedures performed in the GLP facility and every step was initialed and dated by the operator and verifier. The operator and verifier followed specific protocols for Personal Protective Equipment (PPE) and behavior once in the GLP facility. This is a highly-restricted access facility. Due to the importance and quality of work being done only Nebraska Nanomedicine Production Plant employees are granted access. All steps of the approved protocol were documented during manufacturing.

The Quality Assurance Unit was responsible for monitoring this study to assure that the facilities, equipment, personnel, methods, practices, records, and controls were in conformance with the regulations. The Quality Assurance Unit is entirely separate from and independent of the personnel engaged in the direction and conduct of the study. They also maintained a copy of the master schedule sheet, copies of all protocols and determined that no deviations from approved protocols or standard operating procedures were made without proper authorization and documentation.

The balances used two weight standards (10 gram and 200 gram) for calibration checks. The pH probe is standardized using three pH standard buffers (pH 4.0, 7.0 and 10.0) to give a standardized pH slope. All pipets had been calibrated. The Malvern Zetasizer Nano ZSP, which is used for size, polydispersity index and zeta potential measurements, used a 60 nm standard to check calibration. These steps were done before each use.

Equipment such as the Avestin Emulsiflex C3 High Pressure Homogenizer have cleaning protocols which were implemented before and after use. The homogenizer had at least 500 ml of methanol, then sodium hydroxide (NaOH) and lastly 1500 ml of Water For Injection (WFI) circulated through it before and after each manufacturing run. The homogenizer also contains some removable parts which were placed in the oven to bake at 200˚C for at least 60 min. All of the glassware that is used in the GLP facility is also baked at 200˚C for 60 min to remove pyrogens.

Equipment with parts that cannot be removed to be cleaned and sterilized were tested in the following manner. The homogenizer had 40 ml of WFI run through it once it had been cleaned. A sample of this WFI was then tested for endotoxins with the Lonza Limulus Amebocyte Lysate PRYOGEN-500 test kit to verify that the cleaning procedure

was successful. This process was also carried out prior to using the homogenizer to verify no growth of any endotoxin between uses.

UPLC-MS/MS quantitation of MDTG and DTG in monkey plasma and PBMCs

DTG and MDTG concentrations in monkey plasma and PBMCs were determined by UPLC-MS/MS using a Waters ACQUITY H-class UPLC/Xevo TQ-S micro system (Waters, Milford, MA). For plasma analysis, 50 µl of plasma was added to 1 ml of ice-cold LC/MS-grade acetonitrile and 10 µL of internal standard (IS) solution (500 ng/mL each DTG-d3 and myristoylated cabotegravir (MCAB)). For PBMC analysis, 1 mL ice-cold LC/MS-grade acetonitrile was added to pelleted cells (~ 1 X 10^8 cells) and 10 µl IS solution was added. Plasma and PBMC samples were then vortexed for 3 min and centrifuged at 17,000 *g* for 10 min at 4°C. Supernatants were dried using a speed vacuum and reconstituted in 100 µL 50% (v/v) LC/MS-grade acetonitrile in water. Standard curves (0.2 - 2000 ng/mL) of DTG and MDTG were prepared in blank monkey plasma and PBMCs. Chromatographic separation of 10 µL sample was achieved using an ACQUITY UPLC-BEH Shield RP18 column (1.7 µm, 2.1 mm x 100 mm) with a 10-min gradient of mobile phase A (7.5 mM ammonium formate in LC/MS-grade water adjusted to pH 3 with formic acid) and mobile phase B (100% LC/MS-grade acetonitrile) at a flow rate of 0.25 mL/min. The initial mobile phase composition was 40% B for the first 3 min and was increased to 86% B in 0.5 min and held constant for 5 min. Mobile phase B was then reset to 40% in 0.25 min and the column was equilibrated for 1.25 min before the next injection. DTG and MDTG were detected at cone voltages of 10 and 16 volts and collision energies of 25 and 44 volts, respectively. Multiple reaction monitoring (MRM) transitions used for DTG, MDTG, DTG-d3, and MCAB were 420.075 > 277.124, 630.2 > 420.067, 422.841 > 129.999, and 616.277 > 406.094 m/z respectively. Spectra were analyzed and quantified by MassLynx software version 4.1. All calculations were made using analyte peak area to IS peak area ratios.

Table S1. Plasma metabolic panel for animal 5009.

	Day 0	Day 35	Day 77
AST (IU/L)	42	29	34
Alkaline phosphatase (IU/L)	309	222	240
Bilirubin total (mg/dL)	0.2	0.2	0.2
Calcium (mg/dL)	9.6	9.7	9.1
Protein Total (mg/dL)	6.5	7	7.1
Albumin (mg/dL)	4.3	4.3	3.1
Glucose/random (mg/dL)	53	56	51
Urea Nitrogen (mg/dL)	17	22	17
Creatinine (mg/dL)	0.78	0.85	0.89
Bun/creatinine ratio	21.8	25.9	19.1
Sodium (mEq/L)	143	146	145
Potassium (mEq/L)	4	3.7	4.2
Chloride (mEq/L)	106	106	104
Osmolality/calc (mosm/kg)	294	301	297
Carbon dioxide (mEq/L)	26	28	28
Anion Gap (mEq/L)	11	12	13
ALT (IU/L)	29	23	42

Table S2. Plasma metabolic panel for animal 6039.

	Day 0	Day 1	Day 4	Day 7	Day 14
AST (IU/mL)	27	84	64	38	21
Alkaline phosphatase (IU/mL)	105	107	91	98	100
Bilirubin total (mg/dL)	0.2	0.3	0.2	0.2	0.2
Calcium (mg/dL)	9.6	9.5	9.7	9.2	9.3
Protein Total (mg/dL)	6.4	6.6	6.8	6.5	6.5
Albumin (mg/dL)	4.2	4.2	4.1	3.9	3.8
Glucose/random (mg/dL)	66	108	80	76	50
Urea Nitrogen (mg/dL)	16	17	22	17	14
Creatinine (mg/dL)	0.92	0.98	0.85	0.83	1.01
Bun/creatinine ratio	17.4	17.3	25.9	20.5	13.9
Sodium (mEq/L)	145	145	145	146	145
Potassium (mEq/L)	3.8	3.7	4	3.5	3.6
Chloride (mEq/L)	105	106	105	105	106
Osmolality/calc (mosm/kg)	298	301	301	301	296
Carbon dioxide (mEq/L)	25	27	26	27	27
Anion Gap (mEq/L)	15	12	14	14	12
ALT (IU/mL)	20	64	94	67	35

Table S3. Metabolic panel for animal 6049.

	Day 0	Day 1	Day 4	Day 7
AST (IU/L)	29	47	69	38
Alkaline phosphatase (IU/L)	347	356	293	286
Bilirubin total (mg/dL)	0.2	0.3	0.3	0.2
Calcium (mg/dL)	9.1	9.2	9.4	9.2
Protein Total (mg/dL)	6.8	6.9	6.4	6.2
Albumin (mg/dL)	4.3	4.4	4.1	3.9
Glucose/random (mg/dL)	44	69	67	52
Urea Nitrogen (mg/dL)	20	18	20	18
Creatinine (mg/dL)	0.9	1.05	0.98	0.98
Bun/creatinine ratio	22.2	17.1	20.4	18.4
Sodium (mEq/L)	150	147	148	147
Potassium (mEq/L)	3.3	3.4	3.6	3.7
Chloride (mEq/L)	108	106	107	108
Osmolality/calc (mosm/kg)	308	303	305	302
Carbon dioxide (mEq/L)	26	21	25	27
Anion Gap (mEq/L)	16	20	16	12
ALT (IU/L)	17	23	56	57
